# Combined analysis of the salivary microbiome and host defence peptides predicts dental disease

**DOI:** 10.1038/s41598-018-20085-x

**Published:** 2018-01-24

**Authors:** A. Simon-Soro, A. Sherriff, S. Sadique, G. Ramage, L. Macpherson, A. Mira, S. Culshaw, J. Malcolm

**Affiliations:** 1 Genomics and Health Area, FISABIO Foundation (The Foundation for the Promotion of Health and Biomedical Research of Valencia Region), Centre for Advanced Research in Public Health, Avda. De Catalunya, 21/46020, Valencia, Spain; 2 Community Oral Health Section, Glasgow Dental School, School of Medicine, College of Medical, Veterinary and Life Sciences, University of Glasgow, Glasgow, UK; 3 Oral Sciences Group, Glasgow Dental School, School of Medicine, College of Medical, Veterinary and Life Sciences, University of Glasgow, Glasgow, UK; 4 Institute of Infection, Immunity and Inflammation, College of Medical, Veterinary and Life Sciences, University of Glasgow, Glasgow, UK

## Abstract

Understanding the triad of host response, microbiome and disease status is potentially informative for disease prediction, prevention, early intervention and treatment. Using longitudinal assessment of saliva and disease status, we demonstrated that partial least squares modelling of microbial, immunological and clinical measures, grouped children according to future dental disease status. Saliva was collected and dental health assessed in 33 children aged 4 years, and again 1-year later. The composition of the salivary microbiome was assessed and host defence peptides in saliva were quantified. Principal component analysis of the salivary microbiome indicated that children clustered by age and not disease status. Similarly, changes in salivary host defence peptides occurred with age and not in response to, or preceding dental caries. Partial least squares modelling of microbial, immunological and clinical baseline measures clustered children according to future dental disease status. These data demonstrate that isolated evaluation of the salivary microbiome or host response failed to predict dental disease. In contrast, combined assessment of both host response together with the microbiome revealed clusters of health and disease. This type of approach is potentially relevant to myriad diseases that are modified by host–microbiome interactions.

## Introduction

The oral microbiome may offer indicators of both oral and systemic health^1^ and disease^2^. Oral diseases, including dental caries are socioeconomically important, and remain extremely common^3^. In dental caries, changes in the microbiome associate with disease^4^. Previous studies have documented the DNA profile of the bacteria within the carious lesion itself, in the plaque associated with carious teeth^5,6^, and in the saliva^7^. These studies demonstrate some measurable differences in the microbiome, associated with health and disease. However, their findings show variability, likely relating to the age of the participants, the stage of caries development and the site sampled. Development of the oral biofilm is influenced by a variety of factors, including environmental and behavioural factors, and the host immune response to microbial colonisation. It therefore follows that susceptibility to oral disease can be influenced by host immune factors, particularly those found in saliva. Saliva contains a complex mixture of innate antimicrobial proteins and adaptive immune mediators all of which are likely to have a significant impact on the microbial colonisation of the oral cavity, both directly on the microbiome and indirectly by modulating the host^8,9^. Numerous antimicrobials are found in saliva, including the cathelicidins, of which LL37 is the only member in humans, and human neutrophil (alpha) peptides (HNPs)^10^. The antimicrobials found in saliva are produced from a variety of sources, including salivary acini and ducts, oral epithelia and immune cells. Neutrophils are a significant source of antimicrobials in saliva; exemplified by Morbus Kostmann syndrome in which LL37 deficiency manifests as early onset periodontal disease^11^.

Current evidence supports a role for salivary antimicrobials in the development of oral infectious diseases, such as dental caries. Low concentrations of HNPs 1–3 were associated with increased incidence of caries in adolescents^12^. Similarly, low salivary LL37 was associated with higher caries scores in young children^13^ and salivary antimicrobial peptide profiles of infants were found to be significantly different dependent on caries experience^14^. Although *Streptococcus mutans* have long been associated with caries, a small proportion of children with caries do not have detectable *S*. *mutans*^15^, and the relationship between specific bacteria and caries is clearly complex. We previously reported that, in young children, concentrations of salivary antimicrobials were highly correlated within individuals, suggestive of conserved inflammatory stimuli; and we identified a significant positive correlation between the levels of *S*. *mutans* in plaque and the concentrations of salivary HNP-1-3 and LL37^16,17^. There is wide variation in reports of the relationship between LL37 and caries, in part depending on children’s age, and stage of caries^17^. We hypothesized that future caries may be predicted by a combination of host and microbial factors in children; and that combining both host and microbiome would offer better predictive power than either alone. Saliva is an easily sampled diagnostic, populated with bacteria related to the oral biofilms and containing immune mediators generated by the oral tissues. Therefore, we sought to identify the relationship between microbiome and the host response evident in saliva, and clinical disease manifest as caries.

## Results

### Demographics and clinical characteristics

All of the children in the study (N = 33) had a full primary dentition with no missing teeth and no erupted permanent teeth at both time points. Children were grouped according to their caries status throughout the duration of the study: children who were caries-free at baseline and who remained caries-free at the follow-up examination (N-N, n = 14 [Table 1]); children who were caries-free at baseline but developed one or more carious lesions by the follow-up examination (N-Y, n = 5); and children with one or more existing carious lesions at the baseline examination (Y-Y n = 14). At baseline, the caries rate was higher in females compared with boys, although the difference was not statistically significant (13 caries-free males and 6 males with caries compared with 6 caries-free females and 8 females with caries). Once children were separated into groups according to their caries status over time, a significant difference in gender distribution emerged. All of the children who developed caries during the study period were female, such that at the follow-up time point nearly all females in the study had caries, while the majority of caries free children were male (Table 1). We investigated the impact of gender on the mean values of salivary antimicrobials, salivary *S*. *mutans* and microbiome data and found no significant differences according to the gender of children.

**Table 1:** Demographics and clinical characteristics.

		N-N	N-Y	Y-Y	p
		n = 14	n = 5	n = 14	
Mean age					
(years)	Baseline	4.8 (0.29)	4.8 (0.44)	4.5 (0.35)	0.1^a^
	Follow-up	5.7 (0.27)	5.8 (0.38)	5.5 (0.34)	0.1^a^
Dentition					
(n)	Full primary dentition	14	5	14	—
	1 or more permanent teeth	0	0	0	—
Gender					
(n)	Male	13	0	6	
	Female	1	5	8	0.01^b^
Clinical characteristics					
	AMR at baseline	0	0^δ^	2.9 (0.69)^*^	0.0002^a^
	dmft at follow-up	0	1.8 (0.59)	3.9 (0.9)^*^	0.0005^a^
*S*. *mutans*					
(CFE/ml)	Baseline	2.5 (7.1)	4 (31.6)	138 (7.6)	0.34^a^
	Follow-up	5 (6.3)	7112 (2.3)	1667 (4.6)^*^	0.02^a^

Data shown are mean and standard error of the mean unless otherwise indicated.

^a^ANOVA with overall p value reported. Statistically significant differences were followed up with Tukey multiple comparisons: p < 0.05 compared with ^*^N-N or ^δ^N-Y.

^b^Chi-square statistic.

AMR: active carious lesion, missing due to caries or restored due to caries

dmft: number of decayed, missing or filled teeth.

CFE/ml: Colony forming equivalents per ml saliva.

There were no differences in the mean values of biological variables according to gender (data not shown).

At the baseline examination, children with existing caries had higher levels of salivary *S*. *mutans* compared with caries-free children but the difference did not reach statistical significance (Table 1). At the follow-up examination, the numbers of *S*. *mutans* in saliva were highest in the children who developed caries during the study (N-Y) but the difference compared with caries-free children did not reach statistical significance (7112 vs. 5 CFE/ml). The group of children who developed caries during the study had the greatest increase in *S*. *mutans* numbers over time, increasing from a geometric mean of 4 CFE/ml of saliva at baseline to 7112 CFE/ml saliva at follow-up, a 1440-fold increase (Table 1). In comparison, *S*. *mutans* numbers in the N-N group increased only 2-fold from 2.5 CFE/ml at baseline vs 5 CFE/ml at follow-up and 12-fold in the Y-Y group from 138 CFE/ml to 1667 CFE/ml. The increase in mean *S*. *mutans* numbers in the Y-Y group was significantly higher compared with the N-N group at follow-up (Table 1). In this study, all children with caries had detectable *S*. *mutans* as assessed by qPCR.

### Longitudinal changes in salivary microbiome by caries status

**Figure 1:**
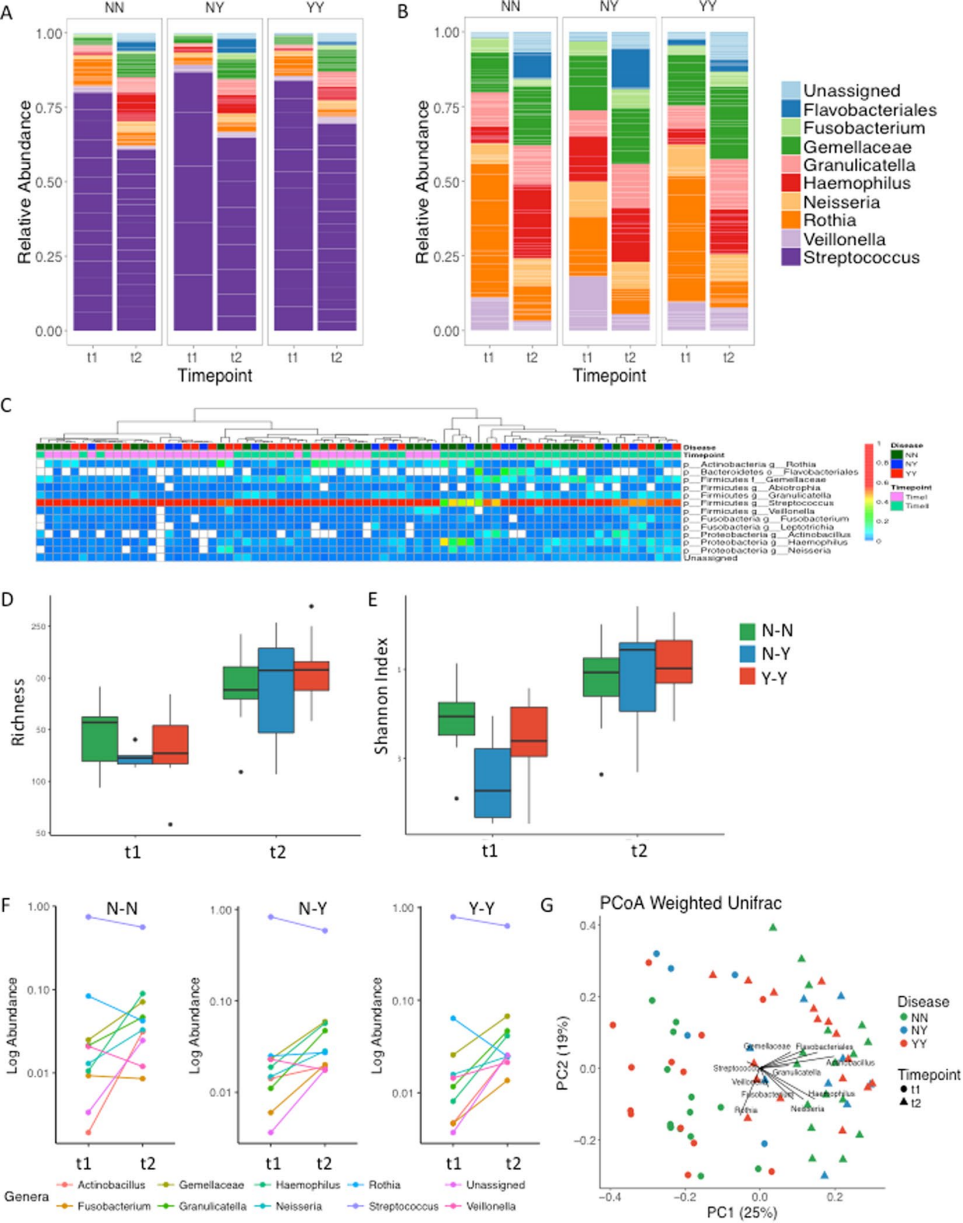
Longitudinal changes in salivary oral microbiome by caries status. (**A** and **B**) Bar graphs indicate the average proportion of each bacterial genus at >1% at baseline (t1) and at the follow-up examination (t2), according to health status: N-N indicates children who remained caries free through the study (n = 14); Y-Y indicates children with caries at both time points (n = 14); N-Y indicates children who were caries-free at baseline and developed caries during the study (n = 5). (**A**) Total bacterial composition. (**B**) Detail of non-*Streptococcus* genera composition. (**C**) Bacterial composition for individual samples. The heatmap shows colour-coded frequencies for all genera found at >1%. Samples are clustered by their genus-level microbial profile. (**D**) Richness, number of OTUs in each group (**E**) Shannon Index of bacterial diversity in each group. (**F**) Line graphs depicting the change in the mean of each genera of >1% abundance at t1 and t2. The panel label N-N, N-Y, Y-Y indicates group. (**G**) Samples occupy a position in this Principal Coordinates Analysis (PCoA) according to their bacterial composition as estimated by 16S rRNA gene pyrosequencing. Unweighted Bray-Curtis distances were used (a weighted analysis provided very similar results). Samples are colour-coded according to the health status of children. Circles indicate t1, baseline; triangles indicated t2, follow-up.

Bacterial composition was estimated by 16S rRNA gene pyrosequencing at both time points (Fig. 1). There were no notable differences in bacterial composition between each of the groups. At both time points, and in all three groups, the bacterial composition was dominated by *Streptococcus* species but with a significant decrease in their proportion at follow-up compared with baseline (A). The N-Y group had significantly higher levels of *Streptococcus* at baseline compared with the N-N group (p = 0.05, Wilcoxon test), but this difference was not maintained at follow-up. Thus, it is plausible that some *Streptococcus* species could be a potential biomarker of caries risk but this could not be confirmed due to the high degree of shared similarity in the 16s gene within *Streptococcus*. There were no notable differences between the groups in the non-*Streptococcus* genera (Fig. 1B). The microbial composition of caries-free and caries-experienced populations overlapped considerably. Over time, there was a significant increase in *Gemella* (Wilcox p-value: 0.0006), *Granulicatella* (p = 0.04), *Neisseria* (p = 0.004) and *Haemophilus* (p = 0.006), at the expense of a significant decrease in *Streptococcus* (Wilcox p-value: 0.0017), observed in the N-N group. Similar trends were also found in the caries-associated groups, although the difference over time in proportion in the N-Y group was statistically significant only for low-frequency genera such as *Gemella*, *Porphyromonas* and *Granulicatella*, showing increased relative proportion from baseline to the follow-up examination. Other genera like *Abiotrophia* appeared to decrease with time in the N-N group, but this trend was not observed in the N-Y and Y-Y groups.

When individual samples were grouped according to their bacterial composition profile (Fig. 1C), they mainly clustered according to time point, and an association based on caries status could not be observed. We searched for potential biomarkers by looking at genera that were present at higher proportions in the N-Y and Y-Y groups compared to the N-N group. These included *Prevotella* and *Actinobacillus*, but the difference was not statistically significant. Nevertheless, there is a trend for some potential biomarkers for health, like the case of *Rothia*. Although some overlap exists between groups, *Rothia* appeared at higher levels in caries-free children with lower levels in N-Y (Fig. 1C). Thus, the potential for some early indicators as biomarkers for health and caries should be tested on larger sample sizes. Given that streptococci are very abundant, and that both health-associated and caries-associated *Streptococcus* species have been identified for this genus, a species-specific analysis with larger sequence reads is required to identify potential disease biomarkers from within this genus.

There was a suggestion of a higher diversity in the N-N group compared to caries-associated groups at baseline, with low-frequency bacteria (those found at <0.1% of the total) accounting for 3.3% of the total, whereas only 0.4 and 0.9% of the total were found in the N-Y and Y-Y groups, respectively. A high microbial diversity has been associated with dental health in other studies^18^. In all groups, over time, there was an increase in community richness as estimated by the number of OTUs (Fig. 1D) and diversity assessed by Shannon Index (Fig. 1E). To illustrate these changes over time, the mean of each genera of >1% abundance was assessed at each time point in each group (Fig. 1F) showing that the majority of genera increased over time, and that there were no notable differences between each of the disease groups. We identified the OTUs that specifically appeared at time 2, and these corresponded to species belonging to the genera *Actinobacillus*, *Gemella*, *Haemophilus*, *Granullicatella*, *Neisseria*, *Fusobacterium* and *Veillonella* (the latter only in the YY group), which therefore seem likely to account for most of the increase in diversity during the time period studied (Fig. 1F).

A global principal component analysis of all the available microbiome data from both time points confirmed that the greatest changes manifest according to the age of children when the samples were collected – with separation of samples according to the time of sampling with baseline samples clustered distinctly from follow-up samples (Fig. 1G). There was no separation between caries groups. To determine whether the weighted analysis influenced the distribution of OTUs, the analysis was repeated using an unweighted method using Bray Curtis distances. This confirmed the samples clustered according to time, and there were no clusters formed on the basis of disease status (data not shown). These data indicate maturation of the microbial populations with increasing age.

### Longitudinal changes in salivary antimicrobials by caries status

**Figure 2:**
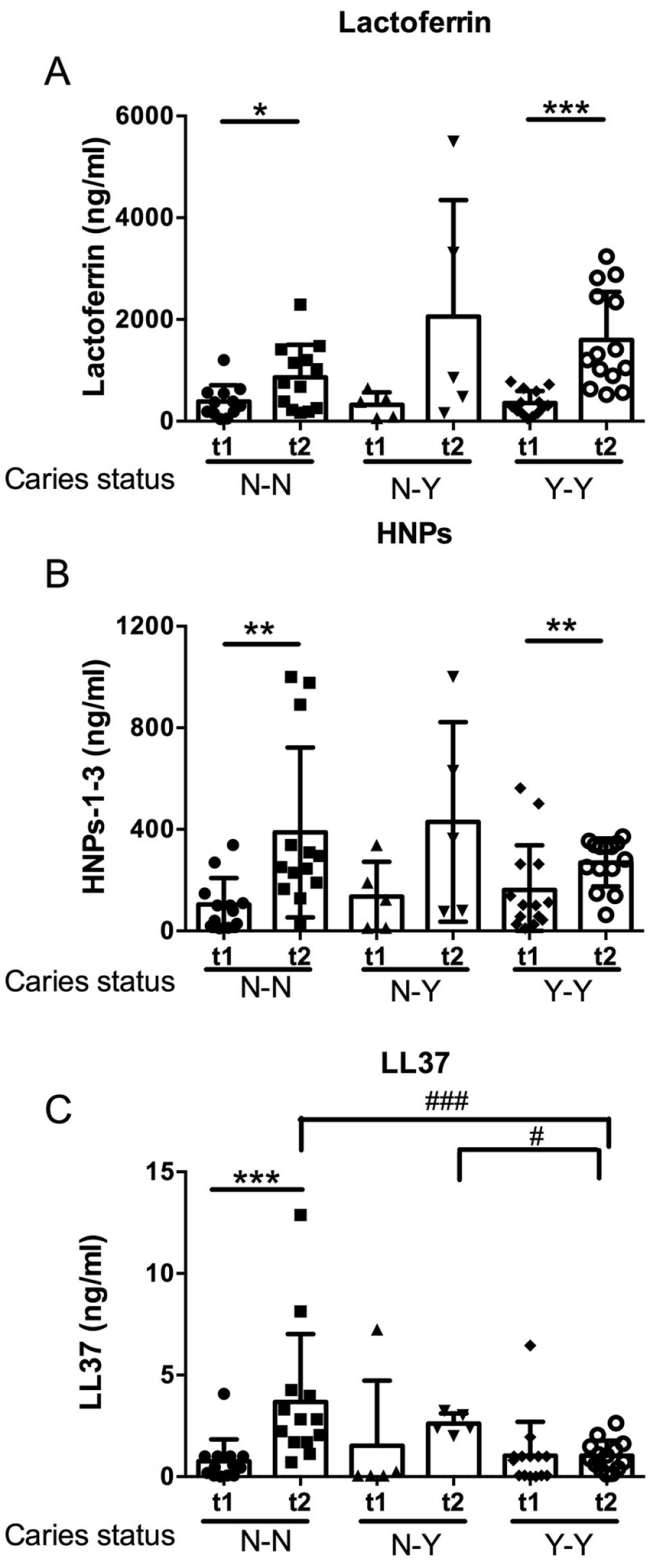
Longitudinal changes in salivary antimicrobials by caries status. Scatterplots comparing the baseline and follow-up concentrations (ng/ml) of (**A**) lactoferrin, (**B**) HNPs-1-3 and (**C**) LL37, grouped according to the change in caries status: N-N (n = 14 children who were caries free at baseline and remained caries free at the follow-up examination), N-Y (n = 5 children who were caries free at baseline but developed caries by the follow-up examination) and Y-Y (n = 14 children who had established caries into dentine at the baseline examination). Each data point represents the mean value for an individual child and the group mean and standard deviation are shown. A statistically significant two-way interaction was achieved for each antimicrobial by time and caries-status (p < 0.05). To test for differences between the concentrations of each antimicrobial at each time point ANOVA with Tukey comparison was used, ^###^p < 0.001 and ^#^p < 0.05. Students *t*-test was used to test the differences between the concentration of each antimicrobial within each caries group ***p < 0.001, **p < 0.01 and *p < 0.5. Raw data are shown. Statistical analyses used log_10_-transformed data.

To assess whether immunological components of saliva might serve as useful biomarkers of caries risk we first examined the concentration of lactoferrin, HNPs-1-3 and LL37 at baseline in each of the caries status groups. The concentrations of each lactoferrin, the HNPs-1-3 and LL37 were similar in each of the groups at baseline (t1, Fig. 2). Indeed, the greatest changes in salivary antimicrobial proteins appeared to manifest over time. Both lactoferrin and the HNPs-1-3 increased significantly from baseline to follow-up in both the N-N and Y-Y groups (Fig. 2A and B). Interestingly, LL37 increased significantly in the N-N group at time 2 compared with time 1 (Fig. 2C) and was significantly higher than the concentration in the Y-Y group at this time. Thus, LL37 increased in the children who remained healthy during the study (N-N) but failed to increase in children with established caries (Y-Y). Taken together, these data suggest that normal maturation of the oral biofilm is accompanied with maturation of salivary antimicrobial responses. A larger sample size would be required to ascertain changes in LL37 in children who develop caries.

### The interaction between salivary microbiome and salivary antimicrobials on caries progression

Antimicrobial components of saliva likely have an impact on the composition of the oral microbial biofilm and LL37 has previously been identified as a potential biomarker of caries risk in older children^13^. Therefore, we investigated the interaction between the oral microbiome and antimicrobial components of saliva to predict caries outcome in our cohort.

**Figure 3:**
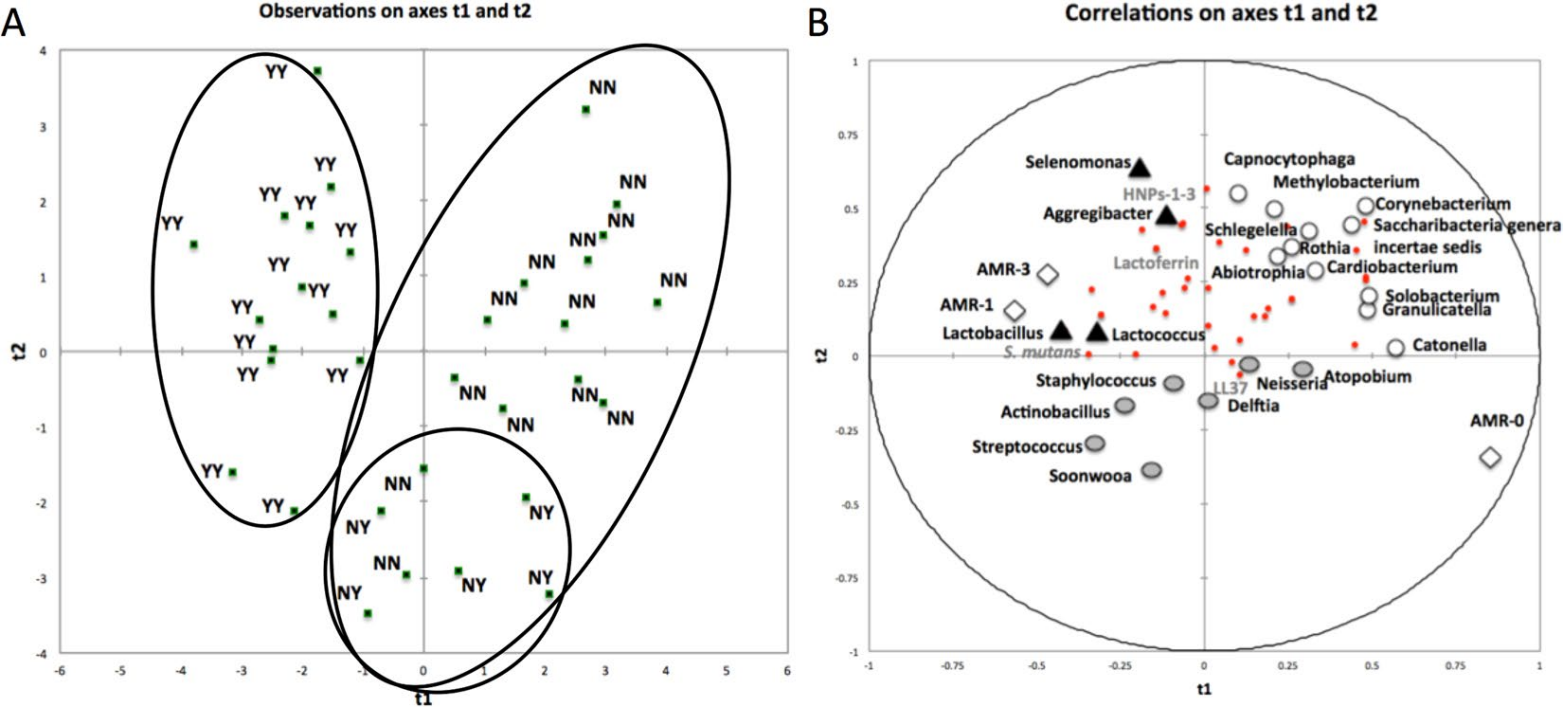
The interaction between salivary microbiome and salivary antimicrobials on caries progression.PLS-DA modeling plots. The PLS-DA model used caries-status at follow-up as the dependent variable with baseline salivary microbiome data, baseline salivary antimicrobial data, baseline *S*. *mutans* qPCR and baseline clinical data as the independent matrix. A model was generated with two significant components R^2^ = 0.623, Q^2^ = 0.207. This model clustered the children into those with established caries (Y-Y) and 2 subgroups of caries-free children, with those who developed caries by the time of the follow-up examination (N-Y) largely distinct from those who remained caries-free at follow-up (N-N) (**A**). PLS loading scatter plot illustrating important variables for clustering. Variables with VIP-values > 1.0 are highlighted: Clinical data at baseline (diamonds), health-associated VIPs in the upper right dimension (open circles), caries-associated VIPs in the upper left (closed triangles). Variables associated with divergence from health and caries are highlighted (grey circles). Small circles represent other variables used in the analysis but not influential for clustering (VIP < 1) (**B**).

The multivariate PLS-DA model used caries status at follow-up as the independent variable with baseline sequencing data, *S*. *mutans* CFE/ml, salivary antimicrobials and baseline AMR scores as the independent matrix. A strong model was generated, with 2 significant components (R^2^ = 0.623, Q^2^ = 0.207). This model clustered the children into those with established caries (n = 14, Y-Y) and 2 subgroups of caries-free children, with those who developed caries by the time of the follow-up examination (n = 5, N-Y) largely distinct from those who remained caries-free at follow-up (n = 14, N-N, Fig. 3A). The first component (t1) correlated positively with the N-N group, with a baseline AMR score of 0 (R^2^ = 0.850) and inversely with in the Y-Y group with a baseline AMR score greater than 1 (R^2^ = −850). Surprisingly, the N-Y group, who also had a baseline AMR score of 0 did not correlate with component 1 (R^2^ = 0.105) and fell between both the N-N and Y-Y groups on this axis (Fig. 3A).

The majority of the very important projections (VIPs > 0.8) were located within the dimension that associated with children who maintained a healthy-status at the end of the study (N-N) and included *Catonella* (VIP 1.508), *Methylobacterium* (VIP 1.366), *Corynebacterium* (VIP 1.441), *Rothia* (VIP 1.226), *Granulicatella* (VIP 1.183) *Capnocytophaga* (VIP 1.146), *Schlegelella* (VIP 1.145), *Cardiobacterium* (VIP 1.122), *Solobacterium* (VIP 1.040), *Abiotrophia* (VIP 1.0) and *Saccharibacteria* generae incertia sedis (VIP 0.966 [clear circles, Fig. 3B]). Meanwhile, the VIPs that correlated positively with the dimension that associated with established caries (and inversely with the health-associated dimension) included *Aggregatibacter* (VIP 1.138), *Lactococcus* (VIP 1.071), *Selenomonas* (VIP 1.058) and *Lactobacillus* (VIP 1.026 and [black triangles, Fig. 3A]). *S*. *mutans* (VIP 0.756), the HNPs-1-3 (VIP 0.461) and lactoferrin (VIP 0.245) were also located within this caries-associated dimension but did not contribute strongly to the model.

The second component (t2) was predominantly defined by strong correlations with several genera of bacteria, most of which were located in the dimension that correlated best with children who maintained a healthy status. These genera included, *Capnocytophaga* and *Corynebacterium* but also *Selenomonas*, which was globally located within the dimension associated with established caries. This component separated the bulk of both the N-N and Y-Y groups from N-Y but did not separate those children who remained healthy from those with established caries. Thus, the N-N and Y-Y groups looked similar on this axis. This observation is in agreement with the bacterial PCA that did not reveal significant differences between health and caries.

Interestingly, LL37 and several genera of bacteria did not correlate with either the health or caries-associated dimensions but instead were located in the dimension that appeared to define the pre-transition from healthy to caries status (N-Y). At baseline, LL37 concentrations were similar across the three groups (Fig. 2C). However, the PLS-DA suggests that even at this early transitional stage of disease, LL37 may interact with certain species of bacteria to modulate caries risk. Further longitudinal studies with a larger cohort of children transitioning to caries are required to confirm this observation.

Both PLS-DA components contributed to the clustering of N-Y from N-N and Y-Y and both components predominantly defined healthy status – with only small deviations from health predisposing to caries onset. Overall these data demonstrate the potential for predicting caries outcome based on multiple biological variables and indicates that these predictions manifest as subtle deviations from health rather than from any overarching influence by one component.

## Discussion

Data reported here demonstrate that complex multi-parameter analysis incorporating the triad of host response, microbiome and disease status, can generate a model to predict disease progression. Moreover, this analysis appeared more sensitive than assessment of components of the triad in isolation.

In both our study and others, longitudinal salivary microbiome analyses, using 454 pyrosequencing, demonstrated marked changes in bacterial composition manifest over time from 3 months to 3 years^19^. Our longitudinal, univariate analyses of microbial composition appeared to mirror these changes. These data suggest that normal maturation of the oral biofilm influences the salivary antimicrobial composition and vice versa. There was a significant increase in LL37 in the children who remained healthy throughout the study, which was not evident in the Y-Y group. This may indicate that these children failed to adequately respond to the increasingly diverse microbial population. Whether this occurred as a consequence of caries or predisposed these children to caries will require further investigation in a larger cohort of children before the onset of established caries.

There was, as we observed before, and as has been observed in other studies, an association between each of the host defence peptides in saliva^17^.

Although the PLS-DA indicated correlations between groups of bacteria with either healthy or caries status, we could not detect any statistically significant differences in the bacterial composition of saliva by caries status at either time point. Previous studies have demonstrated that un-stimulated saliva represents a more diverse bacterial composition than site-specific plaque samples or even stimulated saliva samples^20^, presumably due to bacterial sloughing from different oral niches, including the tongue and other mucosal surfaces and sub and supra-gingival plaque. It therefore follows that subtle changes in bacterial diversity, particularly in site-specific locations, such as a carious lesions, may not be detected by quantification of bacterial diversity in saliva. Despite this limitation, these data are in agreement with a previous study that documented no difference in the bacterial diversity of supra-gingival plaque using 454-pyrosequencing in infants with and without caries^21^. Saliva is a preferable diagnostic/prognostic tool compared with dental plaque as sampling is easier and quicker; however, given the high frequency of *Streptococcus* species in our saliva samples, it is possible that potential bacterial biomarkers other than *Streptococcus* could be masked, and these may be better revealed in dental plaque.

Several oral metagenomics studies, using bacterial PCA, indicate that dental caries, and other oral diseases, are not typically associated with changes in any one bacterial species but rather are associated with polymicrobial shifts that are distinct from health. Previous studies have demonstrated that PLS modelling can improve caries prediction by combining microbial factors associated with dental plaque, salivary bacteria, data from diet diaries, salivary flow rate and buffering capacity and other lifestyle factors in older children^22^. Ideally, with a larger study, we would further cross validate this model by different subsets of subjects according to caries status. As a control validation (and independent question), we carried out the analysis classifying the subjects according to gender, which revealed no prediction of disease. A cross sectional study showed that combined data from dental plaque and mass spectrometry analysis of saliva provided a superior model of caries association than either variable alone^23^. In this longitudinal study, we have used saliva only to provide biological data, combined with disease data to investigate the interaction between bacterial and salivary antimicrobial composition, we used PLS-DA to predict the onset of dental caries. The current analysis, which incorporated aspects of bacterial PCA and included salivary antimicrobial and clinical data, supports the view of polymicrobial shifts from health. Importantly, this analysis provided additional evidence that changes in the salivary antimicrobial composition are also likely important in determining caries outcome. Our PLS-DA identified similarities in the bacterial and antimicrobial composition of saliva of caries-free 4-year-old children prior to the onset of caries, which manifest itself by age 5-years. Individually, our bacterial PCA and univariate analyses of the antimicrobial composition of saliva did not identify changes in children prior to the onset of caries. These data indicate that the interaction between bacterial and antimicrobial composition of saliva is important for determining caries outcome.

Host defence peptides may have therapeutic application. Addition of exogenous lactoferrin restored function in mice genetically deficient in lactoferrin^24^. Potentially, the use of saliva as a diagnostic/prognostic indicator, using multivariate analysis of both host and microbial factors, could allow exogenous host defence peptides or other antimicrobial proteins to be a targeted approach to groups most likely to benefit^25^.

Both the bacterial and antimicrobial composition of saliva can change with the loss of teeth and the eruption of the permanent dentition. Our study benefited from a well-defined cohort, selected based on the absence of any missing or permanently erupted dentition. Furthermore, our longitudinal study design allowed for the grouping of children according to their caries-status throughout the study. Thus, allowing direct comparison of the changes that occurred in children who developed caries with those who remained caries-free and those with established caries. One caveat of this study was the low number of children who remained within the primary dentition and who developed caries during the study period and thus resulting in our small sample size in this group. A further caveat was the gender bias, which arose due to the small sample size. However, this bias did not influence the data presented here. There were no differences in the biological variables according to gender and the PLS-DA analysis was unable to predict the gender of the children using the same predictor variables as reported for the prediction of caries status (data not shown). Despite these caveats, our PLS-DA analysis identified similarities in the bacterial and antimicrobial composition of saliva that may predispose to the development of dental caries. These biological signatures are potentially informative of disease prediction, prevention, early intervention and treatment.

## Methods

### Study design

The participants in this study were selected from a larger study^26^. In brief, at baseline, informed consent was obtained from the legal guardians of 219 children attending 14 preschools in Glasgow. Children with known chronic illness were excluded. Dental exams were carried out and biological samples collected at baseline and follow up. From these, complete data was available for 165 children at baseline. One year later at, follow up, there were 144 with complete data. From this sample of 144 children, we identified 33 children with complete data at both time points, who had not taken antibiotics within the preceding 3 months, who had a complete primary dentition at both times points, with no partially erupted permanent teeth, and no missing teeth, from whom samples of adequate quality were available. Children were grouped into 3 groups according to their caries status throughout the duration of the study: children who were caries-free at baseline and who remained caries-free at the follow-up examination (N-N, n = 14); children who were caries-free at baseline but developed one or more carious lesions by the follow-up examination (N-Y, n = 5); and children with one or more existing carious lesions at the baseline examination (Y-Y n = 14). The study was approved by the West of Scotland Research Ethics Committee 10/SO704/1/62, and carried out in accordance with the Declaration of Helsinki. Dental exams were performed according to The Scottish National Dental Inspection Programme basic inspection criteria, by a dentist calibrated to agreed UK criteria^27^. Teeth were classified according to AMR criteria: sound, active/carious dentinal decay (A), missing due to caries (M) or restored due to caries (R). Thus, as applied to these children, the AMR score equals the number of carious teeth; baseline AMR scores of 0 indicate caries-free children and scores of 1 or more indicates the number of active/carious dentinal decay lesions for that child. Only caries into dentine was recorded, because white spot lesions could not be determined with accuracy outside a clinical setting. Radiographs were not clinically indicated and therefore not available.

### Sample collection

Unstimulated saliva was collected using a child’s oral swab (Salimetrics, Newmarket, UK), which was placed in the mouth and allowed to absorb saliva for 60s. Samples were maintained at 4 °C and processed within 4 h. Saliva was clarified at 6500 × g for 10 min, split into aliquots and stored at −80 °C until use. The cell pellet was retained at −80 °C until required for bacterial DNA extraction.

### Measurement of salivary antimicrobials

The salivary concentrations of calprotectin, LL37, HNPs 1–3 and lactoferrin were estimated by enzyme-linked immunosorbent assay (ELISA; Cambridge Bioscience, Cambridge, UK), according to the manufacturer’s instructions. Briefly, clarified saliva was assayed in duplicate, diluted 1/100 in assay buffer for lactoferrin, and HNPs 1–3 and undiluted for LL37.

### Bacterial DNA extraction and *S*. *mutans* quantification

DNA was extracted using the DNA Masterpure Extraction kit (Epicentre, Hamburg, Germany) following the manufacturer instructions with the addition of a lysozyme treatment at 37 °C for 30 min. *S*. *mutans* DNA was quantified by TaqMan® quantitative PCR (qPCR) using previously described primers and probes specific for *S*. *mutans* gtfB^28^. The number of *S*. *mutans* per ml of saliva was expressed as colony forming equivalents/ml, calculated from a standard curve of DNA extracted from quantified numbers of *S*. *mutans* colony forming units.

### PCR amplification and pyrosequencing

Single-stranded cDNA was constructed with the High-Capacity cDNA Reverse Transcription kit (Applied Biosystems, Grand Island, NY) in 20 µl reactions, with several modifications as previously described^5^. Universal bacterial primers 8 F (5′-AGAGTTTGATCMTGGCTCAG-3′) and 533 R (5′-GCCTTGCCAGCCCGCTCAGGC-3′) were used to partly amplify the 16S rRNA gene from the single-stranded cDNA in two 50 µl reactions, following the PCR and purification conditions previously described^20^. The 500-bp PCR products were purified with the Nucleofast PCR purification kit (Macherey-Nagel, Düren, Germany) and further cleaned by AMPure XP beads (Roche, Basel, Switzerland) before pyrosequencing. The final DNA *per* sample was measured by fluorescence with the Quant-iT PicoGreen dsDNA Assay Kit (ThermoFisher Scientific) in a Modulus 9200 fluorimeter (Turner Biosystems, Sunnyvale, CA, USA) so samples could be mixed in equimolar amounts. PCR products were pyrosequenced from the forward primer end only by using a GS-FLX sequencer with Titanium chemistry (Roche). One-eighth of a plate was used for each pool of 20 samples, which were amplified with a different forward primer containing a unique 8-bp “barcode.”

### Sequence analysis

Reads with an average quality value lower than 20 and/or with more than 4 ambiguities in homopolymeric regions in the first 360 flows were excluded from the analysis. Read ends were trimmed in 10-bp windows if they had a quality value lower than 20. Only reads longer than 200 bp and without mismatches in the primer region were considered. Chimeric sequences were filtered using Uchime and 5.4% of the reads were filtered out as potential chimeras. Singletons were not excluded from the analysis. Sequences were assigned to each sample by the 8-bp barcode and analyzed with the Ribosomal Database Project classifier^29^. Each read was taxonomically assigned down to the genus level with an 80% confidence threshold, and reads giving no bacterial hits were excluded from the analysis. To estimate total diversity, sequences were clustered at 97% nucleotide identity over 90% sequence alignment length UCLUST, and rarefaction curves obtained using the RDP pyrosequencing pipeline. Principal coordinates analysis (PCoA) was performed with FastUnifrac^30^, which compares the shared and unique branches in bacterial communities based on 16S rRNA gene analysis with a phylogenetic approach that takes into account both taxonomically assigned and unassigned reads.

### Statistical analyses

Principal component analyses were used to cluster microbiological data according to caries status. Wilcoxon signed ranks test was applied to test whether two matched samples had the same mean rank. Data were log_10_-transformed to obtain a normal distribution. ANOVA was applied to compare the three group means with Tukey comparison. Two-way ANOVA was used to test the interaction of time and caries-status on salivary antimicrobial protein concentrations. Following a statistically significant interaction (p < 0.05), the differences between each caries group at each time were assessed by ANOVA and the differences over time for each caries group was assessed by Students *t*-test. Partial least squares discriminant analysis (PLS-DA) is a multivariate linear regression analysis that is applicable for the analysis of correlations between dependent variables with both continuous and categorised independent variables, where the independent variables are collinear and the number of independent variables exceeds the number of observations. Mean standardized scores were used for each biological variable input to the PLS-DA and raw clinical data (baseline AMR) were used to predict caries status at the follow-up examination which was entered as the independent variable.

## Data availability

16S rRNA gene sequences are available in the NCBI public repository under Accession Number #SRP127972. The whole datasets generated and analyzed during the current study are available from the corresponding author on reasonable request.
